# The first case of *Streptococcus intermedius* brain abscess with hemophagocytic histiocytosis

**DOI:** 10.1186/s12879-022-07600-2

**Published:** 2022-07-18

**Authors:** Jia Zhang, Jianjun Wang, Jing Gan, Rong Luo, Xiaolu Chen

**Affiliations:** 1 Department of Pediatrics, West China Second University Hospital, Sichuan University, No. 20, Section Three, South Renmin Road, Chengdu, 610041 China; 2 Key Laboratory of Obstetrics & Gynecologic and Pediatric Diseases and Birth Defects of the Ministry of Education, Sichuan University, Chengdu, Sichuan China; 3 Key Laboratory of Development and Maternal and Child Diseases of Sichuan Province, Chengdu, 610041 China

**Keywords:** Infection-associated hemophagocytic syndrome (IAHS), *Streptococcus intermedius*, Brain abscess, Metagenomic next-generation sequencing (mNGS), Case report

## Abstract

**Background:**

Hemophagocytic lymphohistiocytosis (HLH) is a rare but potentially life-threatening immune syndrome associated with an excessive systemic inflammatory response. Viral infection caused HLH is the most common secondary HLH, but there are relatively few reports of HLH caused by bacterial infection. The present study is the first case of HLH caused by Streptococcus intermedia meningitis.

**Case presentation:**

The patient is an 11-year-old and 9-month-old boy. The main symptoms are fever, headache, and vomiting. The imaging finding of the brain is cerebritis and brain abscess. The cerebrospinal fluid (CSF) routine test showed increased nucleated cells, but the smear and culture of CSF were negative. The metagenomics next-generation sequencing (mNGS) of CSF detected *Streptococcus intermedius*, and the body temperature of the children returned to normal after antibiotic treatment according to etiology. One week later, the child developed fever again, with Kawasaki disease-like manifestations. After high-dose immunoglobulin therapy, the body temperature returned to normal again. The routine blood test showed a progressive decrease in leukocytes and platelets, and bone marrow biopsy detected histiocytes phagocytosed blood cells. Then infection-associated hemophagocytic syndrome (IAHS) was diagnosed, high-dose methylprednisolone and sequential therapy were given and the patient’s recovery was encouraging.

**Conclusions:**

Our case shows that HLH can also be secondary to *Streptococcus intermediate* infection, and early bone marrow biopsy is the golden standard for HLH diagnosis. mNGS can improve the detection sensitivity for pathogens when traditional pathogenic tests are negative. Conventional chemotherapy regimens may not be required for IAHS when high-dose glucocorticoids and immunoglobulin therapy are effective.

## Background

Hemophagocytic lymphohistiocytosis (HLH) is a syndrome characterized by an excessive systemic inflammatory response. HLH may have a genetic cause or may also be secondary to an infection, autoimmune disease, malignant tumors, immunodeficiency, etc. [1]. The pathogenesis of HLH is currently believed to be a multisystem inflammatory response resulting from defective granule-mediated cytotoxicity mediated by natural killer cells (NK cells) and cytotoxic T lymphocytes (CTL). Hypercytokineemia is the most important pathological feature of HLH [2]. Infection-associated hemophagocytic syndrome (IAHS) is an infectious agent-induced HLH that can result from acute infection based on genetic susceptibility, and most cases occur in immunocompromised patients, often with multiple complications, leading to delayed diagnosis and high mortality. This study reports on the development of HLH following *Streptococcus intermedius *(*S. intermedius*) brain abscess infection in a previously healthy patient.

## Case presentation

**Fig. 1:**
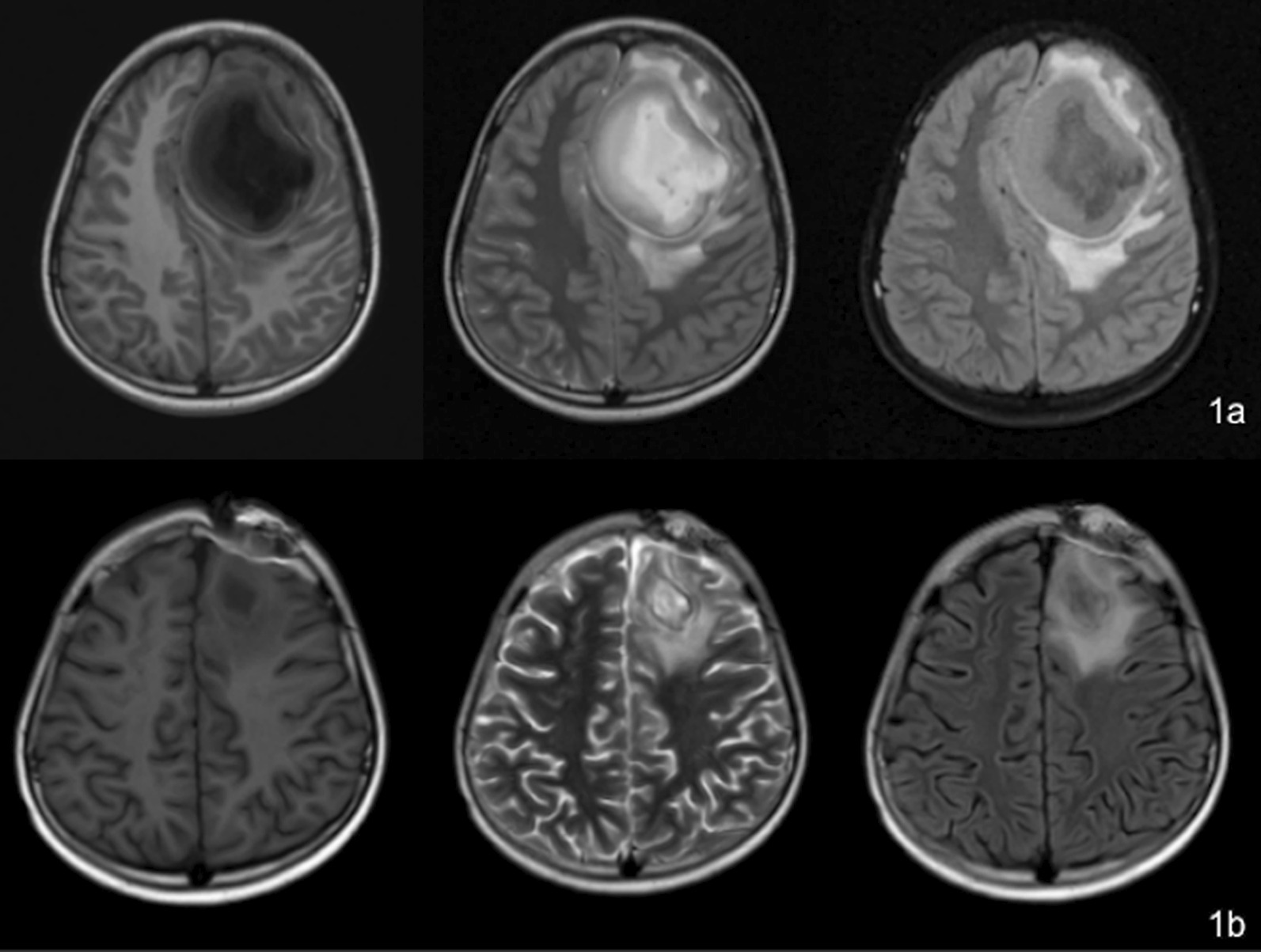
**a** Head MRI before the brain abscess showed cystic occupation of the left frontal lobe. **b** Head MRI after excision of brain abscess showed the occupying effect in the left frontal lobe was significantly reduced

The patient who is male, 11 years and 9 months old, was admitted to our hospital on June 7, 2021, due to recurrent fever, headache, and vomiting for 11 days. On May 25, 2021, the patient underwent deep supratentorial abscess resection and craniotomy decompression in another hospital. Physical examination on admission: The mental response was normal. Dark red rashes about the size of pinpoints were on the neck, trunk, and upper limbs. Mild lumbosacral tenderness, and no abnormality in the cardiopulmonary, abdominal, and nervous system examination. No history of repeated infections or immunodeficiency. One month before admission, the patient developed vomiting and fatigue. The vomiting was progressive aggravation and projectile. Cranial MRI showed a left frontal lobe brain abscess (Fig. 1a). Laboratory findings after admission are as following: blood routine (day 1) test: WBC 12.3 × 10^9^/L, N 69.0%, L 23.9%, HGB 120 g/L, PLT 361 × 10^9^/L, CRP 10.7 mg/L. Liver and kidney function, electrolytes, disseminated intravascular coagulation, cellular and humoral immunity were all normal. CSF routine test (day 2): nucleated cells were 495 × 10^6^/L, 83% were neutral lobulated, 17% were lymphocytes. CSF biochemistry: protein 819.4 mg/L, glucose 0.98 mmol/L, chloride 127.5 mmol/L, lactic dehydrogenase 143 U/L. Pathogens test in CSF: cytomegalovirus was positive, smear and culture were negative. EEG (day 4) was normal. Crainal CT (day 5): brain abscess in left frontal lobe after the operation, which was smaller than before. After admission, ceftriaxone, ceftazidime combined with vancomycin were given for anti-infection, and the child’s temperature was still abnormal. The second CSF routine test (day 6): nucleated cell count was 990 × 10^6^/L, neutral lobulation was 86.0%; biochemical: protein 754.5 mg/L, sugar 0.96 mmol/L, LDH 191 U/L; smear and culture were negative. Serum cytomegalovirus IgG 100 U/mL, IgM, and DNA were negative; EBV IgM, PCR were normal. The cellular/humoral immune screening was unremarkable. The patient’s clinical presentation suggested bacterial intracranial infection, while no pathogenic bacteria were identified, we then performed metagenomic next-generation sequencing (mNGS) on cerebrospinal fluid, patient’s CSF was collected and the DNA was extracted and purified. After obtaining the sequencing data, human reads were removed by mapping reads to human reference genome. The remaining data were aligned to the microbial genome database. The database collected microbial genomes from NCBI. It contains more than 20,000 microorganisms, including 11,910 bacteria, 7103 viruses, 1046 fungi and 305 parasites. Finally get the microbial compositions of the sample. And the mNGS of CSF (day 6) detected *S. intermedius*. The antibiotic was adjusted to penicillin and combined with vancomycin for anti-infection. After that, the temperature gradually returned to normal, and vancomycin was gradually reduced. Head enhanced MRI (day 10): Changes after brain abscess resection of deep supratentorial lesions: Partial bone defect in the left frontal lobe, cystic mass in the left frontal lobe, the uneven signal in capsule, with surrounding. The brain parenchyma was obviously edematous, and the cyst wall was obviously thickened and enhanced unevenly (Fig. 1b). No abnormality was found in the enhanced MRI of the spinal cord. CSF (day 12): nucleated cell count 300 × 10^6^/L, lymphocytes 62.0%, neutral lobulation 26.0%; protein 632.4 mg/L, sugar 1.16 mmol/L, LDH 50 U/L; smear and culture was normal.

**Fig. 2:**
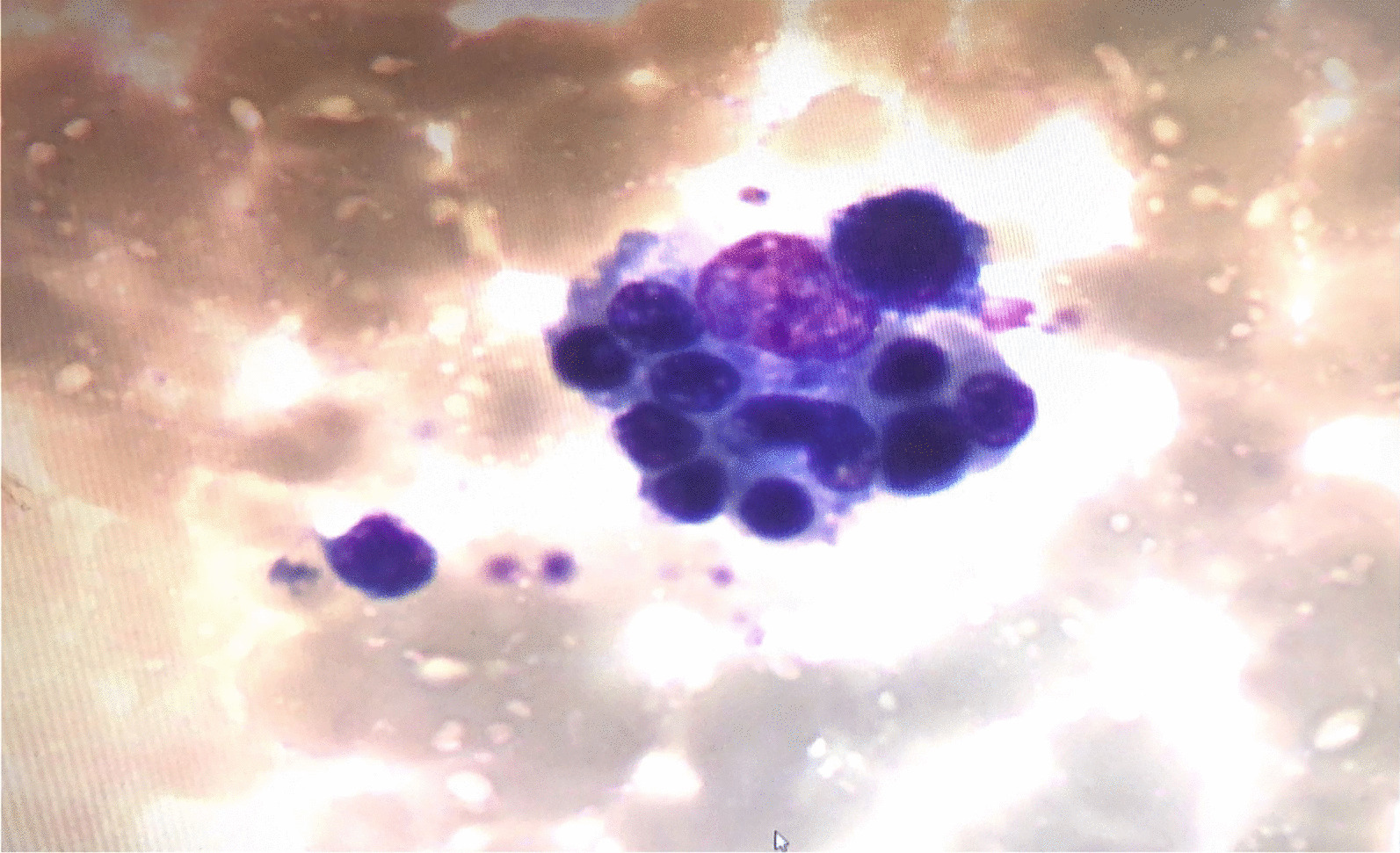
The examination of marrow cells showed that histiocytes phagocytosed blood cells

One week after the penicillin treatment, the patient’s body temperature returned to normal but rose again very soon. Blood routine test (day 22): WBC 3.4 × 10^9^/L, N 54.4%, HGB 131 g/L, PLT 197 × 10^9^/L, CRP 8.8 mg/L. Procalcitonin: 0.14 ng/mL. CSF routine test (day 22): nucleated cells were 25 × 10^6^/L, 84.0% were lymphocytes, 12.0% were neutral lobes, 4.0% were monocytes. CSF biochemistry: protein 565.5 mg/L, glucose 1.78 mmol/L, chloride 126.0 mmol/L, LDH 9 U/L, ADA 1.8 U/L. Pathogens tests for CSF and blood were all negative. Cranial CT scan performed on 30th June showed improvement of brain abscess. At this time, the child developed a skin rash, enlargement of bilateral cervical lymph nodes, conjunctival hyperaemia, red and cracked lips, and strawberry-like tongue, followed by swelling of both hands and feet, without hepatosplenomegaly. As Kawasaki disease was suspected, a single high-dose (2 g/kg) intravenous administration of immunoglobulin (IVIG) was given. And the patient’s temperature returned to normal. Blood routine test showed leukopenia and thrombocytopenia (WBC 1.1 × 10^9^/L, ANC 0.25 × 10^9^/L, N 22.7%, L 66.4%, HGB 123 g/L, PLT 74 × 10^9^/L). Liver function indicated elevated liver enzymes and triglycerides (ALT 127 U/L, AST 778 U/L, TG 9.2 mmol/L), hypofibrinogenemia (Fg 70 mg/dL), significantly elevated ferritin (> 16,500 ng/mL). Cytokines level: IL-1β 30.8 pg/mL (< 5 pg/mL), IL-2 1683 U/mL (223–710 U/mL), IL-10 10.70 pg/mL (< 9.1 pg/mL), TNF-α 20.4 pg/mL (8.1 pg/mL). NK cell activity assay and cytotoxic degranulation assay were normal. Bone marrow biopsy found histiocytes phagocytosing blood cells (Fig. 2). Considering the diagnosis of hemophagocytic syndrome due to brain abscess as the most likely possibility, the patient was treated with intravenous methylprednisolone (20 mg/kg/day) for 3 days followed by oral prednisolone in a gradually tapering dose. Blood routine test on July 21, showed WBC 5.9 × 10^9^/L, N 51.7%, ANC 3.05 × 10^9^/L, HGB 119 g/L, PLT 264 × 10^9^/L. ALT was 13 U/L, AST was 26 U/L. Triglycerides was 1.43 mmol/L. Serum Ferritin was 96.80 ng/mL. Cranial MRI on July 23: Compared with the results of June 16, the brain lesions in the left frontal lobe were significantly reduced, the brain tissue edema was significantly reduced, and residual lesions were found in the left frontal lobe. To explore the potential genetic cause of HLH in this patient, a targeted next generation sequencing (NGS) panel was applied, including LYST, CTPS1, PIK3CD, PRF1, SRGN, CD27, LAMP1, ARF6, GZMB, RAB27A, BLOC1S6, CORO1A, UNC13D, STXBP2, GNLY, STK4, PRKCD, AP3B1, ITK, STX11, CARD11, MCM4, MAGT1, SH2D1A, XIAP, and IL2RG genes. And a missense variant (c.600 A>C) in the gene IL2RG was detected. According to ACMG guidelines, the variant was considered a variant of uncertain significance (VUS). Large insertions, deletions, and duplications were not detected. After discharge, the child was given oral prednisone and was followed up for about half a year. Prednisone was gradually reduced and finally stopped after half a year. The symptoms of HLH have not recurred, and the blood routine test was normal.

## Discussion and conclusion

Hemophagocytic syndrome (HPS), also known as hemophagocytic lymphohistiocytosis (HLH), is a syndrome of the excessive inflammatory response caused by primary or secondary immune abnormalities. Infection-associated HLH (IAHS) is the most common type of secondary HLH. A nationwide study in Japan showed that 90% of children with HLH belong to IAHS [3], including viral, bacterial, fungal, and parasitic infections, which can manifest as infection opportunistic pathogenesis upon induction and/or compromise of host immunity. Viral infection is the most common cause of reactivation, both in healthy people and in immunosuppressed patients, of which EB virus infection is the most common cause. HLH caused by bacterial infection is relatively rare, with a small number of case reports so far. In Japan, the incidence of childhood HLH caused by bacterial infection accounts for about 6% of all IAHS cases [3], including *Streptococcus pneumonia*, *Mycobacterium tuberculosis*, Leptospira, Brucella, and group B hemolytic streptococcus, *Streptococcus suis*, *Haemophilus influenzae*, *Klebsiella pneumonia*, *Staphylococcus aureus*, *Mycobacterium bovis*, *Salmonella typhimurium*, *Mycoplasma pneumonia*, Q fever, Jungle typhus, etc. [4–9]. There is no report of *S. intermedius* brain abscess-induced HLH mentioned in literature before.

*Streptococcus intermedius* is a facultatively anaerobic, microaerobic, and B-hemolytic Gram-positive microorganism that usually inhabits the oral, throat, and gastrointestinal flora and is an opportunistic pathogenic bacteria. The clinical association of *S. intermedius* with abscess propensity has long been recognized and is the main etiology of brain abscesses [10, 11]. Al Masalma et al. reported that 25% of brain abscesses were associated with *S. intermedius* infection [12]. *S. intermedius* brain abscess formation begins with human fibronectin and laminin-binding and subsequent IL-8-mediated tissue damage from monocytes, which activates neutrophil chemotaxis and contributes to pro-inflammatory cytokines accumulate, leading to brain tissue damage [11]. Intemedilysin (ILY) and NanA are the main pathogenic factors, which can secrete proteolytic enzymes, damage tissues, promote abscess formation, and increase the chance of infection when the body’s immune system is weakened. Therefore, *S. intermedius* predisposes to severe brain and liver abscesses without bacteremia [13, 14]. Dental caries, tooth extraction, otitis media, sinusitis, cyanotic congenital heart disease, etc., are risk factors for *S. intermedius* infection [15]. *S. intermedius* meningitis is mainly characterized by intermittent fever and persistent headache. When a persistent headache occurs, attention should be paid to the formation of brain abscess. *S. intermedius* is currently sensitive to β-lactam drugs, and subgingival isolates are sensitive to both amoxicillin and clindamycin [16]. The most commonly used antibiotic regimen for *S. intermedius* meningitis is a combination of ceftriaxone and metronidazole [11]. Of course, treatment should be guided by the epidemiology of local pathogens and the results of bacterial culture and drug susceptibility testing. Abscess drainage and surgery remain the only interventions to limit abscess expansion.

The current accepted diagnostic criteria for HLH were revised by the Histiocyte Society in 2004 [20], and HLH can be diagnosed if one of the following two criteria is met: (1): Molecular diagnosis is consistent with HLH, and there are currently known HLH related pathogenic genes. (2): Meets 5 of the following 8 indicators: ① Fever: body temperature > 38.5 ℃, lasting for > 7 days; ② Spleen enlargement; ③ Cytopenia (involving two or three peripheral blood lineages) : hemoglobin < 90 g/L, platelet < 100 × 10^9^/ L, neutrophil < 1.0 × 10^9^/L and not caused by decreased hematopoietic function of bone marrow; ④ Hypertriglyceridemia and/or hypofibrinogen: triglyceride > 3 mmol/L or higher than 3 SD of age, fibrinogen < 1.5 g/L or lower than 3SD of age; ⑤ Blood macrophages were found in the bone marrow, spleen, liver, or lymph nodes; ⑥ Serum ferritin ≥ 500 ug/L; ⑦ Decreased or absent NK cell activity; ⑧ sCD25 (soluble IL-2 receptor) was elevated. The diagnosis of IAHS depends on the comprehensive judgment of clinical manifestations, laboratory tests, and clinical experience. Serum ferritin (SF) level can be used as an important diagnostic power, and hemophagocytic cells founded in bone marrow are the gold standard of HLH. In a retrospective study, HLH was diagnosed in 61% of patients with SF level above 100,000 ng/mL [17], and SF level was associated with mortality and disease outcome in patients with HLH [18, 19]. Although the child in this study had no fever after anti-infection and glucocorticoid treatment, without enlargement of liver and spleen, and NK cell activity assay and CD-107a level were normal, the early elevated SF level (> 16,500 ng/mL) and the bone marrow biopsy confirmed the diagnosis of HLH. Therefore, it is recommended that SF screening and bone marrow biopsy, if necessary, be performed for leukopenia and/or erythrocytopenia and/or thrombocytopenia (≥ 2), even in the absence of hepatosplenomegaly. In this case, the condition of the patient with HLH secondary to *S. intermedius* brain abscess, got improved after penicillin and high-dose methylprednisolone therapy. The pathogenesis and clinical manifestations of IAHS are complex, and the condition varies greatly. Early diagnosis and individualized treatment are the keys to improve the prognosis. Therefore, appropriate selection of treatment for the primary disease is important for IAHS therapy, and on this basis, the pediatric HLH-2004 treatment plan should be used as a reference [20]. For patient with stable condition and mild clinical symptoms, glucocorticoids can be used in addition to anti-infection and symptomatic treatment. If the situation cannot be improved, additional chemotherapy drugs such as cyclosporine A (CSA) and etoposide (VP16) can be considered.

HLH frequently occurs in patients with inherited defects in genes related to the cytotoxic pathways of T and NK cells, or in genes involved in Epstein Barr virus (EBV) clearance, and infection frequently serving as a trigger for clinical manifestation. The serum IL2 level in this patient was significantly increased, and the hemophagocytic syndrome gene screening detected a missense mutation c.600 A>C in the IL2RG gene. IL2RG gene pathogenic variant was often the cause of severe combined immunodeficiency, but this patient did not have repeated severe infection previously and did not have peripheral blood lymphopenia and cellular humoral immunodeficiency. According to ACMG guidelines, this mutation is a variant of unclear significance, so correlation with the IL2RG gene mutation and performance of hemophagocytic syndrome in this case is uncertain. However, it cannot be excluded that the child is a carrier of IL2RG mutation. In addition, the routine etiological tests of this patient were all negative, and *S. intermedius* was detected by mNGS of CSF. Previous research showed that the sensitivity of mNGS in the diagnosis of encephalitis and meningitis was 73% and the specificity was 99%, which could increase the positive rate of pathogen detection by 13.1% [21, 22]. mNGS for CSF detection is an unbiased calculation of microbial DNA fragments and has nothing to do with bacterial survival. The detection accuracy, especially after anti-infection therapy, is beneficial improved for early diagnosis and timely treatment [23]. Due to the high sensitivity of mNGS detection, it is necessary to pay attention to the false positive results. In this patient, the CMV-PCR result was positive in CSF, while CMV-IgM antibody and CMV-DNA copy number in serum were negative. In addition, without any antiviral therapy, CMV-PCR result in CSF was negative 4 days later, and mNGS test for CMV was negative too. So it was considered that the initial positive CMV-PCR of CSF was probably false positive, which might be related to cross contamination of target sequence or amplification.

In conclusion, the case we present shows that *S. intermedius* brain abscesses can induce HLH. Although the diagnosis of IAHS can sometimes be very difficult, we recommend always using HLH as the differential diagnosis, especially in case with cytopenias, and bone marrow biopsy is the gold standard for diagnosis of HLH. At present, mNGS becomes a advantage technology of pathogen identification in CNS infection disease. mNGS can improve the pathogen detection sensitivity when the traditional etiological test is negative, which is convenient for early and accurate diagnosis and treatment. With the advancement in diagnostic technology, the application of mNGS in pathogen identification in CNS infection will be further improved to be more effective. Conventional chemotherapy regimens may not be required for IAHS treatment when high-dose glucocorticoids and immunoglobulin therapy is effective. Early diagnosis and individualized treatment are key factors in improving IAHS prognosis.

## Data Availability

Not applicable. All data generated or analyzed during this study are included in this published article.

## Abbreviations

HLH: Hemophagocytic lymphohistiocytosis
CSF: Cerebrospinal fluid
mNGS: Metagenomics next-generation sequencing
IAHS: Infection-associated hemophagocytic syndrome
NK: Natural killer cell
CTL: Cytotoxic T lymphocytes
*S. intermedius*: *Streptococcus intermedius*
IVIG: Immunoglobulin
NGS: Next generation sequencing
VUS: Variant of uncertain significance
HPS: Hemophagocytic syndrome
ILY: Intemedilysin
SF: Serum ferritin
CSA: Cyclosporine A
VP16: Etoposide
EBV: Epstein Barr virus
